# Multilevel log linear model to estimate the risk factors associated with infant mortality in Ethiopia: further analysis of 2016 EDHS

**DOI:** 10.1186/s12884-022-04868-9

**Published:** 2022-07-26

**Authors:** Solomon Sisay Mulugeta, Mitiku Wale Muluneh, Alebachew Taye Belay, Yikeber Abebaw Moyehodie, Setegn Bayabil Agegn, Bezanesh Melese Masresha, Selamawit Getachew Wassihun

**Affiliations:** 1 Department of Statistics, College of Natural and Computational Sciences, Debre Tabor University, Debre Tabor, Ethiopia; 2 Department of Statistics, College of Natural and Computational Sciences, Mekdela Amba University, Mekane Selam, Ethiopia

**Keywords:** ZINB, AIC, BIC, DIC, R, EDHS, Multilevel log linear, Infant mortality

## Abstract

**Background:**

Infant mortality is defined as the death of a child at any time after birth and before the child’s first birthday. Sub-Saharan Africa has the highest infant and child mortality rate in the world. Infant and child mortality rates are higher in Ethiopia. A study was carried out to estimate the risk factors that affect infant mortality in Ethiopia.

**Method:**

The EDHS− 2016 data set was used for this study. A total of 10,547 mothers from 11 regions were included in the study’s findings. To estimate the risk factors associated with infant mortality in Ethiopia, several count models (Poisson, Negative Binomial, Zero-Infated Poisson, Zero-Infated Negative Binomial, Hurdle Poisson, and Hurdle Negative Binomial) were considered.

**Result:**

The average number of infant deaths was 0.526, with a variance of 0.994, indicating over-dispersion. The highest mean number of infant death occurred in Somali (0.69) and the lowest in Addis Ababa (0.089). Among the multilevel log linear models, the ZINB regression model with deviance (17,868.74), AIC (17,938.74), and BIC (1892.97) are chosen as the best model for estimating the risk factors affecting infant mortality in Ethiopia. However, the results of a multilevel ZINB model with a random intercept and slope model revealed that residence, mother’s age, household size, mother’s age at first birth, breast feeding, child weight, contraceptive use, birth order, wealth index, father education level, and birth interval are associated with infant mortality in Ethiopia.

**Conclusion:**

Infant deaths remains high and infant deaths per mother differ across regions. An optimal fit was found to the data based on a multilevel ZINB model. We suggest fitting the ZINB model to count data with excess zeros originating from unknown sources such as infant mortality.

## Introduction

Infant mortality is the death of infants under the age of one. Infant mortality is measured by the infant mortality rate (IMR). It refers to any death occurring after birth but prior to the child’s first birthday. Infant mortality rate is an indicator of national health because it is linked to a number of factors including maternal health, health-care quality and access, socioeconomic conditions, and public health practices. The socioeconomic status of the family has a greater impact on infant survival than any other age group in the population [1–3].

In 2020, 2.4 million children died in their first month of life worldwide. Every day, approximately 6700 newborns die, accounting for 47% of all child deaths under the age of five, up from 40% in 1990. The number of newborn deaths worldwide has decreased from 5 million in 1990 to 2.4 million in 2020. However, the decline in neonatal mortality between 1990 and 2020 was slower than the decline in neonatal mortality among children under the age of five. In 2020, neonatal mortality rate were highest in Sub-Saharan Africa 27 (25–32) deaths per 1000 live births), followed by Central and Southern Asia, which had 23 (21–25) deaths per 1000 live births [4]. Children born in Sub-Saharan Africa are ten times more likely than children born in high-income countries to die within the first month of life. In 2020, nationally infant mortality rates will range from 1 to 44 per 1000 live births, with the risk of babies dying before the age of 28 days being about the lowest in the world. It happened 56 times [5, 6].

Worldwide, 50 % of the deaths occurred in six countries, including Ethiopia, and 1500 children died every day. The majority of children died from preventable or treatable causes such as birth complications, pneumonia, diarrhea, neonatal sepsis, and malaria [7, 8]. Every day, more than 704 children die from diseases that could be avoided. If current trends continue, more than 3,084,000 children will die by 2030 [9]. There have been regional variations in child mortality in Ethiopia. Child mortality rates in Ethiopia range from 39 per 1000 live births in Addis Abeba to 125 per 1000 live births in Afar. Infant mortality rates are higher. Infant mortality risks are higher during the first few months of life in particular. This also holds true in Sub-Saharan Africa [10]. According to the 2016 Ethiopian Demographic and Health Survey (EDHS), the infant mortality rate for the 5 years preceding the survey was 48 deaths per 1000 live births. Nearly 72% of under-five deaths occur before the age of one in our country. The magnitude of infant mortality varies greatly across the country [11].

To estimate the risk factor of infant mortality, various countries conducted studies [12–16]. There have been many small-scale surveys that investigated a specific set of variables. The analyses in these studies looked at binary logistic and survival factors [16–19]. While binary logistic regression undercounts the total number of deaths since multiple deaths are rolled into a single unit to meet the criteria for binary logistic regression, it provides sufficient information to study multiple child deaths. As a method of analysis, count regression was chosen in this study. The response variable is the number of infant deaths (count), and the main objective is to estimate how this number changes with the increase in explanatory variables. Modeling count data using Poisson regression is the most widely known method. Despite its underlying assumption of equality in distribution (i.e., the mean and variance are equal), its use is limited in some real-world cases with uneven distribution (i.e., the variance is greater than the mean or smaller than the mean). In the event of excessive variation, parameter estimates, standard errors, tests, and confidence intervals may be distorted. Over dispersion has a variety of causes. Excessive zero counts or censorship are two examples. Over-dispersed count data are common in many fields, prompting the development of statistical methodologies for modeling over-dispersed data [20, 21]. The negative binomial distribution resembles the Poisson distribution, except that it has a longer, fatter tail when the variance greater than the mean. Depending on the degree of over dispersion, the negative binomial model may capture more zeros than the Poisson model [22, 23]. Conversely, the model may be insufficient for empirical applications with zero values in the data. By allowing over dispersion, zero inflated models can model the excessive proportion of zero values. When the number of zeros is large, it performs better than the Poisson or negative binomial models [24]. Studies on infant mortality often used prevalence alone, Chi square associations, or logistic and survival modeling [15, 25–29]. Logistic regression divides the dependent variable into two categories, dead or alive, undercounting the rate of infant mortality. Using this approach, multiple child deaths will be considered as one death for the purpose of a logistic regression. A multi-index count model involves a two-part model with extensive (zero) and intensive (positive) margins, representing nonoccurrence and occurrence of death, respectively. Combining the zero-point mass distribution and the nonzero-point distribution results in a finite mixture, an alternative to the two-part process. As a solution, we proposed a two-part random effects model for infant mortality data. This study uses Multilevel Log Linear Modeling to estimate the risk factors for infant mortality in Ethiopia. A model’s main strength is that it accounts for survey design features such as clustering, and stratification, which could lead to inaccurate statistical estimations.

### Contribution

As far as we know, this is the first study to use multilevel count regression models such as ZINB at a multi-regional level to assess the impact of various covariates on infant mortality. Furthermore, the estimation of the specific risk factors with infant mortality will help to prioritize interventions and reveal patterns that will help to improve outcomes. Finally, to provide researchers with detailed information about how to use over dispersed, under dispersed, and zero inflated regression models.

## Methods

### Study design, area and sampling

**Fig. 1:**
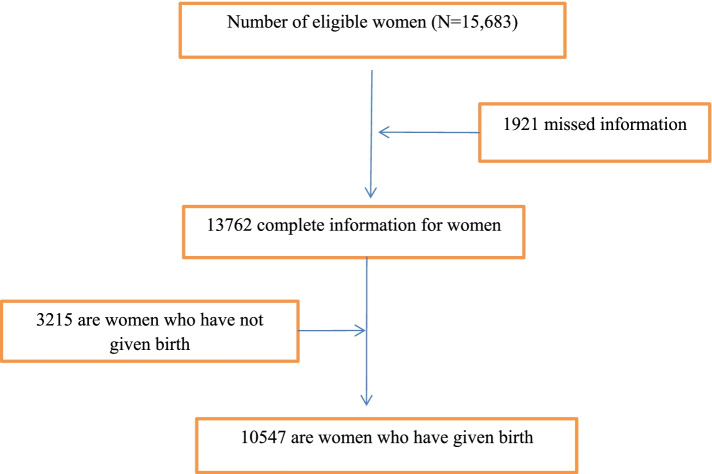
Sample selection procedure

For this study, a cross sectional secondary data from the Ethiopian Demographic and Health Survey (EDHS) of 2016 were used. In response to a reasonable request, and after ensuring that the data was licensed, survey information was downloaded from the DHS website. EDHS 2016 is a part of the global level DHS venture that is funded by the USA agency for global development (USAID) and carried out by using the Ethiopian vital Statistical enterprise. The DHS is accomplished each 5 years, and the 2016 survey is Ethiopia’s fourth, overlaying all nine areas and two administrative city [11]. The Ethiopian Demographics and Health Survey (EDHS) program collected data from nationally representative samples of all age groups and key indicators. The survey collected data on socio-demographic, socioeconomic, and maternal variables. The stratified two-stage cluster sampling method was used to select study participants. In the 2016 survey, a total of 645 EA (202 urban, 443 rural) were chosen. The survey included 15,683 women of childbearing age and 18,008 households in these census areas. The Ethiopian Demographics and Health Survey Report contains detailed sampling methods, which are available on the Measure DHS website (www.dhsprogram.com) [11]. In this study, a total weighted sample of 10,547 mothers from reproductive age mothers who were interviewed about infant deaths in the preceding 5 years before the survey were included in this analysis for this study (Fig. 1).

### Study variable

#### Dependent variable

The number of infant deaths per mother, which refers to the death of an infant before his or her first birthday, was the dependent variable.

### Independent variable

Possible predictors of infant death use such as region, mother’s age, father’s education level, mother’s education level, father’s occupation, mother’s occupation, family size, mother’s age at first birth, religion, source of water, toilet facility, wealth index, Sex of child, Marital status, Contraceptive use, birth order, preceding birth interval, child twin, birth weight, place of delivery antenatal visit, breastfeeding, and residence were potential predictors of infant death.

### Statistical software

SPSS software version 23 was used for recording the secondary data, which was exported to R software version 3.4.1 and analyzed with R software.

### Statistical models

Sometimes, the response of interest in epidemiological studies involves a count; such as number of infant deaths. In these instances, a multilevel count regression model is most appropriate due to the nature of the data set. A multilevel count regression model can account for lack of independence across levels of nested data (in this case, individual mothers nested within regions). Multilevel models used to assess whether the effects of predictors vary from region to region. A number of multilevel count regression models are applied to this study, including Poison, Negative Binomial, Zero-Inflated Poisson, Zero-Inflated Negative Binomial, Hurdle Poisson, and Hurdle Negative Binomial.

### Multilevel Poisson regression model

In practice, although the value of goodness of fit tests as measured by R Square indicates that the model can adequately explain the response variable, there is a violation of the assumption that such problems as heteroscedasticity and dependence error exist. The multilevel Poisson regression model for a count *Y*_*ij*_ for *i*^*th*^ individual mothers in *j*^*th*^ region can be written as:

$${y}_{ij}\left/ {\lambda}_{ij}\right.= poisson\left({m}_{ij},{\lambda}_{ij}\right)$$

The standard link function for the Poisson distribution is the logarithm, and we get

$${\mu}_{ij}=\ln \left({\lambda}_{ij}\right)$$

$${\mu}_{ij}=\ln \left({\lambda}_{ij}\right)={\beta}_0+{\sum}_{h=1}^k{\beta}_h{X}_{hij}+{u}_{oj}+\sum_{i=1}^k{u}_{ij}{X}_{hij},\kern1.5em \mathrm{h}=1,2,3,\dots, \mathrm{k}\kern1.75em$$

The first part of the equation, ${\beta}_0+{\sum}_{h=1}^k{\beta}_h{X}_{hij},$ is called the fixed part of the model. The second, ${u}_{oj}+\sum_{i=1}^k{u}_{ij}{X}_{hij},$ is called the random part. Where, *λ*_*ij*_= the expected/mean number of death for *i*^*th*^ mother in *j*^*th*^ region, *X*_*hij* = *x*1*ij*, *x*2*ij*, *x*3*ij*, …, *xkij* _ represent the first and the second level covariates, *β* = *β*_0_, *β*_1_, *β*_2_, …. *β*_*k*_ are regression coefficients, *u*_0*j*_, *u*_1*j*_, *u*_2*j*_, …. . , *u*_*kj*_ , are the random effect of model parameter at level two (region level) and assume a distributed with means zero and has a multivariate normal distribution with a constant variance matrix [30, 31].

### Multilevel negative binomial regression model

NB regression model is a popular approach to analysis over-desperation data. Often, because of the hierarchical study design or the data collection procedure, over-desperation and lack of independence may occur simultaneously, which render the standards NB model inadequate. Multilevel negative binomial regression model is given by [32]:

$$\Pr \left({Y}_{ij}={y}_{ij}\right)=\frac{\varGamma \left({y}_{ij}+v\right)}{y_{ij}!\varGamma (v)}\ X\ \frac{v^v{\mu_{ij}}^{\ast {y}_{ij}}}{{\left(v+{\mu_{ij}}^{\ast}\right)}^{v+{y}_{ij}}},\kern2em {y}_{ij}=0,1,2,\dots \dots \dots$$

With mean and variance given, respectively, as follows:

$$E\left({Y}_{ij}\right)={\lambda_{ij}}^{\ast }=\log \left({\mu}_{ij}\right), and\ \mathit{\operatorname{var}}\left({Y}_{ij}\right)={\lambda_{ij}}^{\ast }+\alpha {\left({\lambda_{ij}}^{\ast}\right)}^2$$

Where

$${\mu}_{ij}={\beta}_{0j}+{\beta}_{1j}{X}_{1 ij}+{\beta}_{2j}{X}_{2 ij}+\dots \dots \dots +{\beta}_{kj}{X}_{kij}$$

### Multilevel ZIP regression model

When the non-zero part is a discrete random variable, a popular approach to analyze count data with excess zeros is to use a zero-inflated Poisson (ZIP) regression model. Often, because of the hierarchical study design or the data collection procedure, zero inflation and lack of independence may be present simultaneously as a con-sequence of the inherent correlation structure and underlying heterogeneity. A multi-level ZIP regression model developed to handle correlated count data with extra zeros. A multi-level ZIP regression model incorporating random effects to account for the data dependency [31, 33]. Consider the hierarchical state where *Y*_*ij*_ for *i*^*th*^ individual mothers in *j*^*th*^ region. The observations may be taken to be independent between regions, but certain within-household and within-individual correlations are anticipated, which can be modelled explicitly through random effects attached to the linear predictors:

$$\log \left({\lambda}_{ij}\right)={X_{ij}}^{\prime}\beta +{X_{ij}}^{\prime }U$$

$$\log \left(\frac{\varnothing_{ij}}{\left(1-{\varnothing}_{ij}\right)}\right)={\xi}_{ij}={Z_{ij}}^T\gamma +{V}_{0j}+\sum_{h=1}^k{V}_{hj}{Z}_{hij}$$

Here, the covariates *X*_*ij*_ and *Z*_*ij*_ appearing in respective Poisson and logistic components are not necessarily the same and *β* and *γ* are corresponding vectors of regression coefficient [34, 35].

### Multilevel zero-inflated negative binomial (ZINB) regression model

Count data with excess zeros and dispersion occur in many areas. Although zero-inflated negative binomial (ZINB) regression is useful for modeling such data, over dispersion, zero-inflation, and correlation may occur concurrently due to the hierarchical study design or the data collection procedure [34, 36]. A multilevel ZINB regression incorporating random effects to account for data dependency and over-dispersion is used. Let *y*_*ij*_(*i* = 1, 2, …, *m*; *j* = 1, 2, ……, *n*_*i*_) be a count say, the death of the *i*^*th*^ infant in the *j*^*th*^ province/region. Then the two level ZINB regression model can be expressed in vector form as:

$$\log \left(\frac{\varnothing }{\left(1-\varnothing \right)}\right)=\xi = A\alpha +{R}_ww+{R}_ss$$

$$\log \left(\mu \right)= X\beta +{R}_uu+{R}_vv$$

Where, A, X, *R*_*w*_, *R*_*s*_, *R*_*u*_, *and R*_*v*_ are design matrices. For simplicity of presentation, the random effects *w*, *s*, *u*, *and v*, are assumed to be indipendant and normally distributed with mean zero and variance *δ*_*w*_^2^, *δ*_*s*_^2^, *δ*_*u*_^2^, *and δ*_*v*_^2^,respectively [34].

### Multilevel hurdle regression model

The Poisson-hurdle model is composed of two sub models in such a way that the total likelihood is decomposed into two separate likelihoods that can be maximized independently. The proportion of zero counts is described by a binary model with covariate dependence, which is commonly modeled with the logit link, but the probit and log–log links are also used. Second, the positive counts are represented by a zero-truncated Poisson distribution. Thus, the Poisson-hurdle model is a non-overlapping mixture of two Poisson distributions: a degenerate Poisson with mean zero and a truncated Poisson defined on positive integers [37]. A popular approach to the analysis of such data is to use a Hurdle Poisson regression model [38]. Let *y*_*ij*_(*i* = 1, 2, …, *m*; *j* = 1, 2, ……, *n*_*i*_) be a count say, the death of the *i*^*th*^ infant in the *j*^*th*^ province/region The probability that a particular count occurs at the *j*^*th*^ region for the *i*^*th*^ infant under the Poisson-hurdle model is given by:

$$\Pr \left({Y}_{ij}=0\right)={p}_{ij}$$

$$\Pr \left({Y}_{ij}=k\right)=\left(1-{p}_{ij}\right).\frac{e^{-{\mu}_{ij}\left(\frac{{\mu_{ij}}^k}{k!}\right)}}{1-{e}^{-{\mu}_{ij}}}$$

Further, the probabilities *p*_*ij*_ and the means of the truncated Poisson distribution, *μ*_*ij*_, might depend on covariates. In the Poisson-hurdle model each individual belongs to the one of the two components of the mixture, i.e. zero-part or truncated Poisson part. The repeated measures Poisson-hurdle model implies that the same individual might belong to the zero part at one time point, while it can change the class at another visit, e.g. when a positive count is observed. Therefore, the repeated measures Poisson-hurdle model is defined for each time point [37].

### Parameter estimation and model comparison

The most popular methods used to estimate the parameter of a count regression model is maximum likelihood estimation technique (MLE). MLE estimation technique determine the parameters by maximize likelihood of the sample data. MLE methods are versatile and apply to most models and to different types of data [32]. Model comparison was made using deviance information criteria (DIC), Akaike’s Information Criterion (AIC), and Bayesian’s Information Criterion (BIC) [30].

## Result

A total of 10,547 women were included in the study, with 7570 (71.8%) of the mothers having no infant deaths and 18 (0.2%) having lost 6 infants. Further examination of the number of infant deaths revealed that the variance (0.994) is greater than the mean (0.526), indicating over-dispersion. This indicates that the data could be better fitted by count data models that account for excess zeroes (Table 1).

**Table 1:** Frequency distribution of number of infant death per mothers

Number of infant deaths	Frequency	Percentage (%)
0	7570	71.8
1	1346	12.8
2	992	9.4
3	435	4.1
4	134	1.3
5	52	0.5
6	18	0.2
Total	10,547	
Mean	0.526	
Variance	0.994	
Skewness	2.18	
Kurtosis	5.133	

### Socio-demographic and economic characteristics of infant death per mother in Ethiopia

Somalia has the highest (0.69) mean infant death per mother, while Addis Abeba has the lowest (0.089). Rural areas had a higher mean number of infant death per mother (mean = 0.593) than urban areas (mean = 0.23). The infant death per mother with a primary education is (0.57), which is higher than for mothers with a secondary or higher education (0.184). Similarly, the average number of infant deaths for the poorest mothers (0.65) is higher than for the richest mothers (0.34). As a result, a woman living in a better and standard economic level had a lower mean number of infant deaths than a woman living in a high income economic level. Male infant death per mother is higher (0.53) than female infant mortality. Infants born with birth orders five and above had the highest mean (0.95) number of deaths. The infant death rate per mother is high for infants born at more than one interval of birth (0.43). It was also discovered that families who did not use toilets had the highest mean number of infant deaths per mother, which was around 0.6(Table 2).

**Table 2:** Characteristics of variables with infant deaths per mother in Ethiopia, 2016 EDHS

			Infant deaths per mother	
Variable	Categories	N	mean	variance
Region	Tigray	1023	.3675464	.6553841
	Afar	1043	.6759348	1.315226
	Amhara	972	.5308642	.8239692
	Oromia	1569	.5066922	.9095534
	Somali	1496	.6858289	1.370127
	Benishangul Gumuz	867	.6597463	1.434902
	SNNPR	1269	.5602837	1.017854
	Gambela	703	.4082504	.6236925
	Harari	603	.4245439	.7795959
	Addis Adaba	460	.0891304	.1249361
	Dire Dawa	542	.4778598	.8451651
Residence	Rural	8579	.5929596	1.102893
	Urban	1968	.2322154	.4122407
Mother education level	Primary	9427	.5662459	1.055737
	Secondary and above	1120	.1839286	.3414752
Use of toilet	No use	4696	.6005111	1.074027
	Use toilet	5851	.4655614	.9213352
Economic status of house hold	Poorest	5710	.6472855	1.218713
	Middle	1453	.4886442	.8216685
	Richest	3384	.3362884	.6270481
Current marital status	Married	9817	.5402873	1.018777
	Other	730	.3287671	.6160437
Birth order	First	2152	.1277881	.2900317
	2-4	4624	.3611592	.6889172
	+ 5	377	.9543888	1.46211
Birth interval	One	7162	.4286512	.7741992
	> 1	3385	.7308715	1.39652
Sex of child	Male	5433	.527517	.9963432
	Female	5114	.5236605	.9911279

### Model selection criteria

The results in Table 3 were used to identify the appropriate count model among the six commonly considered count models using AIC, BIC, and Deviance statistics. A lower value for any of these criteria indicates a better fit or that the model is a parsimonious model. Therefore, multilevel ZINB regression model has a lower deviance, AIC, and BIC value. As a result, we conclude that the multilevel ZINB regression model fits the data better than the other count regression models (Table 3).

**Table 3:** Model selection criteria for the multilevel count regression models

Criteria	Poisson	NB	ZIP	ZINB	HP	HNB
-2*LL	19,318.35	18,433.94	17,961.52	17,868.74	18,941.83	18,927.8
AIC	19,360.35	18,481.94	18,025.52	17,938.74	19,023.83	19,015.8
BIC	19,512.9	18,656.27	18,257.27	18,192.97	19,321.64	19,335.39

*AIC* Akaike’s information criterion, *BIC *Bayesian information criterion, *HNB *hurdle negative binomial, *HP *Hurdle Poisson, *NB *negative binomial, *ZINB* zero-infated negative binomial, *ZIP* zero-inflated Poisson

### Risk factors associated with infant mortality in Ethiopia

When counting infant deaths per mother, there are often excess zeros. In such cases, zero-inflated Poisson regression (ZIP) and zero-inflated negative binomial regression (ZINB) can be used, but zero-inflation and correlation may occur simultaneously because of hierarchical study design. It is still possible to use ZIP or ZINB to overcome these challenges. In this study, we overcome these problems through multilevel ZINB regression. To account for both the hierarchical structure of the dataset and excessive zeros, a ZINB model with random effects (mixed ZINB) was used as shown in Table 4, and two random effects parameters were estimated. Compared to mothers who live in rural areas, the expected log count for infant death for mothers who live in urban areas was 1.22 (exp (0.2)) times the expected log count for mothers who live in rural areas. These findings indicate that the risk of infant death decreases with each additional year a mother has lived at the time of giving birth. Consequently, it follows that if the age of the mother at the time of birth increases by one unit, the log count of infant deaths will decrease by 0.98 (exp (− 0.022)). As well, for each unit increase in age at first birth, the expected log count of infant mortality decreased by 0.97(exp(− 0.03)). The expected log count of infant mortality for infants born to birth order 5 and above is 5.16(exp(1.641)) times that of infants born to birth order 1. It has been estimated that infant mortality is 2.45 (exp(0.9)) times higher among those born between 2 and 4 than those born first. In comparison with breast-feeding mothers, the expected log count of not breast-fed faced a higher mortality rate 1.433(exp(0.36)) than the expected log count of mothers who breastfed their babies. Compared to mothers without contraceptive use, the expected log count of infant death is 0.86 (exp(− 0.2025)) times lower. In comparison to single birth mothers, the expected log count of infant deaths was 1.37 (exp(0.314)) times higher with multiple birth mothers. Additionally, through the use of inflated models, it was shown that the odds of the infant mortality rate per mother being zero when living in an urban area were 1.584(exp(0.46) times higher than when living in rural areas. The estimated odds of infant deaths with non-breastfed mothers were 1.433(exp(0.36)) times higher than those with breastfed infants (Table 4).

**Table 4:** The results of random coefficients ZINB model on infant mortality in Ethiopia, 2016 EDHS

	Estimation of Fixed effect count part			
	Estimate	Std. Error	z -value	***P***-value
**Intercept**	−1.154	.1755	−6.576	< 0.0001 ***
**Residence (Rural)**				
Urban	0.2	0.08867	−2.201	0.027721 *
**Mother Current age**	− 0.022	0.004055	−5.352	< 0.0001 ***
**Age of first birth**	−0.026	0.005595	− 4.551	< 0.0001 ***
**Birth Weight**	−0.0013	9.842e-05	− 13.448	< 0.0001 ***
**Breast feeding (Yes)**				
No	0. 36	0.04715	7.592	< 0.0001 ***
**Source of water (piped)**				
Other	0.008403	0.04378	0.192	0.847788
**Toilet facility (No use)**				
** Use**	0.05693	0.04008	1.420	0.155504
**Economic status (Poorest)**				
Middle	− 0.1204	0.06285	−1.916	0.055332
Richest	− 0.16	0.05931	− 2.641	0.0083 **
**Contraceptive use (No)**				
Yes	− 0.203	0.05555	−3.644	0.0003 ***
**Marital status (Marriage)**				
Other	− 0.082	0.1210	− 0.681	0.496096
**Father education level (No education level)**				
Primary	− 0.08	0.03956	− 1.964	0.0495 *
Secondary & above	−0.114	0.06314	−1.802	0.071536
**Father occupation (NO)**				
Working	0.073	0.05306	1.373	0.169737
**Mother occupation (No)**				
Working	0.04	0.04157	0.863	0.388187
**Birth order (**First)				
2-4	0.9	0.07762	11.360	< 0.0001 ***
≥5	1.64	0.09742	16.843	< 0.0001 ***
**Place of delivery (home)**				
Health facility	0.062	0.05.192	1.190	0.234101
**Birth index (with index 1)**				
>1	− 0.41	0.03654	− 11.205	< 0.0001 ***
**Sex of child (Male**)				
Female	0.022	0.03176	0.704	0.481214
**Type of birth (Single)**				
Multiple	0.314	0.07262	4.319	< 0.0001 ***
**Women education level (No education)**				
**Primary**	− 0.1181056	0.0635275	− 1.859	0.063010
Secondary & above	− 0.02046	0.09384	− 0.218	0.827404
**House hold size**	0.063	0.01630	3.888	0.0001***
**Estimation of Fixed effect for zero inflated part**				
	**Estimate**	**Std. Error**	**z-value**	**Pr(>|z|)**
**Intercept**	−1.34512	0.26289	− 5.117	< 0.0001 ***
**Place of residence (Rural)**				
Urban	0.45816	0.20217	2.266	0.0234 *
**Breast feeding (yes)**				
No	1.36809	0.20337	6.727	< 0.0001 ***
**Economic status (Poorest)**				
Middle	0.15264	0.16295	0.937	0.3489
Richest	0.18216	0.14271	1.276	0.2018
**Contraceptive use (No)**				
Yes	0.18384	0.14174	1.297	0.1946
**Marital status (Marriage)**				
Other	0.25808	0.32338	0.798	0.4248
**Mother occupation (No)**				
Working	− 0.20082	0.11136	− 1.803	0.0713
**Place of delivery (Home)**				
Health facility	− 0.02153	0.13604	−0.158	0.8742
**Estimation of Random effect truncated count part**				
	**Variance**	**Std. error**		
Community (Intercept)	8.03802	2.8351		
Residence	0.03726	0.19303		
Age of mother	0.01822	0.1350		
House hold size	0.03562	0.1887		

Std.Error=Standard error

*= significant at *P*-value < 0.05

** = significant at *P*-value <0.01

***= significant at *P*-value <0.001

## Discussion

A multilevel log linear model was used in this study to estimate the risk factors related to infant mortality in Ethiopia, using a 2016 EDHS dataset. Estimating these risk factors are useful for policymakers in identifying improvements to the lives of people and tracking progress toward achieving the MDGs. The results of this study shed light on the causes of infant mortality in Ethiopia. Compared to infants living in rural areas, the study estimated odds that the infant mortality rate per mother would become zero when the infant was in an urban area. It is evident from this result that urban mothers experience a lower infant mortality rate than their rural counterparts. This result in lined with the previous study that [13, 39–41]. Infant mortality is a major issue, even in rural India [42], the location of the infant’s residence (rural versus urban) may also affect their health. In rural areas, lacking infrastructures such as electricity, running water, toilets, and basic health care facilities could cause infants to become seriously ill. The problem stems from the lack of adequate health facilities in rural areas. As a result, rural areas do not have the same level of infrastructure as urban areas, which prevents mothers from accessing better health care in urban areas.

According to the age of the mother at the time of birth, infant deaths are more likely to occur when the age of the mother at birth is younger than an increase in the mother’s age. Both the mother and the child may be at risk of being in danger when many births occur at a young age This finding is supported by a study conducted in low- and middle-income countries [43], Ethiopia [42], Kenya [13], and Nepal [41] which found that both teenage and elderly maternal ages predispose infants to increased mortality risks during infancy, with the risk being higher for teenage mothers. Additionally, researchers found a higher risk of death for children whose mothers were younger, confirming previous research findings that suggest child mortality can be influenced by birth age in a number of ways [43–46]. Most importantly, the covariate age of the mother at a time of birth was discovered to significantly increase the risk of death of an infant born at a young age of the mother and there is evidence that prematurity increases the risk of infant mortality among mothers [42, 43]. The increased risk of child death among younger mothers is due to immature reproductive systems and less stability in dealing with the complexities of childbirth.

This study also discovered that household size has a significant impact on the number of infant deaths. The risk of infant death increased significantly as household size increased. Then the covariate household size was also identified statistically that infants born to mother live with large family members have more likely to die, this study in line with [17, 25, 47–49], infants born to mothers with a large household size were more likely to die than infants born to mothers with a small household size. Too many siblings at home may result in insufficient and improper care for infants. The infant death is often correlated with infant birth weight. For every unit increase in infant birth weight, the expected number of infant deaths decreases. This study supported the previous study [13, 50, 51]. Smaller than average sized infants are at a higher risk than larger than average sized infants because their bodies are weaker, making them more susceptible to illness that could kill them later in life. Possibly, decreasing birth weight increases the risk of infant mortality [43]. The highest mortality risk is associated with births of order six or higher with a short preceding interval. There is a greater mortality risk for infants born to mothers with birth orders second and above than for infants born to mothers with birth orders one. It indicates that as birth order increases the likelihood death of infant death become wide large. The variable birth interval has a significant effect on infant mortality, however. This study was consistent with the done by [13, 23, 25], and shows that the risk of mortality was higher for children born in a higher birth order. It is evident that the child death rate falls with increasing birth intervals. The chance that a higher income mother will produce a healthy child is greater, however the number of infant death is lower. The findings of this study were consistent with previous research [21–26], which found that infants born to poor mothers are at a higher risk of dying than those who are richest. Perhaps it is because these mothers can afford good nutrition for their babies and have time to spend with them.

According to this study, infants who have not been breastfed face a greater risk of death than mothers who are breastfeeding. This study supported a previous study [52, 53], which suggested that the risk of death for infants who were not breastfed was higher than for those who were breastfed. It is probable that breastfed babies are protected from childhood illnesses [42]. The risk of infant mortality is reduced when parents are more educated. It confirms the finding from the previous study: the higher the level of education of the parents, the lower their infant death [13, 23, 43, 54, 55]. This is because more educated parents are more aware of the importance of providing better care for their child and his wife during the antenatal and postnatal periods. Moreover, his study found that the risk of an infant death from a contraceptive-using mother was significantly lower than the death of an infant from a non-contraceptive-using mother, which agrees with previous research findings, use of contraceptive is the issue for reducing infant mortality and birth interval [17, 23]. In a similar study, vaccinated infants had a lower risk of mortality than non-vaccinated infants [48]. This could be because those mothers who use contraception seek advice from a physician in a health or non-health institution. A study we conducted found that multiple-birth infants are at greater risk of death than single-birth infants. Having multiple births is associated with numerous negative birth outcomes, which are linked to a higher rate of infant mortality. The risk of infant mortality is higher with multiple births than with single births; similar to previous studies, this study determines that birth type affects the risk of infant mortality [23, 25, 56]. Multiple births increase the economic burden of the family, which can lead to poor nutrition and health care for the infant.

### Strengths and limitations

Although the EDHS is a nationally representative, large-scale dataset, there may be some limitations to this analysis. Participants were asked to recall events from the 5 years before the survey, and they may have forgotten some details. Another limitation is the cross-sectional nature of the data, which prevents the identification of causal relationships between outcome and exposure variables. As well, since this study is based on self-reported values taken from a nationally representative survey, it is subject to several sources of error: estimates for specific countries may change over time as a result of these limitations, and these should be taken with caution. However, the EDHS is a nationally representative dataset and is designed and deployed rigorously by the Centers for Disease Control and Prevention using a global framework. This allows the results to be generalized throughout the country, even with these limitations. Due to the use of similar instruments across countries, there will also be an opportunity to compare the results internationally.

## Conclusion

This study aimed to estimate the risk factors associated with infant mortality in Ethiopia using multilevel log linear model. As a result, we discovered an excess of zeroes in the dataset as well as several sources of heterogeneity. This study included 10,547 mothers, and the results show that 71.8% of the mothers did not experience infant death, while 0.2% experienced at least 6 infant deaths per mother in their lifetime prior to the survey. Multilevel Poisson model analysis determined ZINB to be most parsimonious to estimate the risk factors associated with infant mortality in Ethiopia. According to the findings, the number of infant deaths per mother varies by region. Residents’, age of the mother at the time of birth, household size, mother’s age at first childbirth, breast feeding, child’s birth weight, contraceptive use, birth order, wealth index, father education level, multiple birth, and birth interval were found to be significant risk factors for infant mortality. The findings of this study suggest that, more efforts are needed to expand educational programs to educate mothers about the advantages of contraception, breast feeding, and spacing birth intervals in order to reduce infant death per mother.

## Data Availability

The datasets used in this study were obtained from a freely available public dataset that does not identify survey participants. A data set is available on https://dhsprogram.com/data/available-datasets.cfm. Approval was sought from MEASURE DHS/ICF International and permission was granted for its use.

## Abbreviations

AIC: Akaki Information Criteria
BIC: Bayesian information criteria
CSA: Central Statistical Agency
EAs: Enumeration areas
EDHS: Ethiopian Demographic and Health Survey
EFD: Exponential family distribution
HP: Hurdle Poisson
HNB: Hurdle negative binomial
IMR: Infant mortality rate
LRT: log likelihood ratio test
MDG: Millennium Development Goals
MLE: Maximum likelihood estimation
NB: Negative binomial
PR: Poisson regression
ZINP: Zero-inflated Negative binomial
ZIP: Zero-inflated Poisson
SDG: Sustainable Development Goal
WHO: World Health Organization
